# How to Create Trusted Tribological Characterization Data of Soft Polymers as Input for FEM Simulations?

**DOI:** 10.3390/ma16010131

**Published:** 2022-12-23

**Authors:** Marin Herr, F. Xavier Borras, Dirk Spaltmann, Mirco Kröll, Franz Pirker, Ulrike Cihak-Bayr

**Affiliations:** 1 AC2T research GmbH, Viktor-Kaplan-Straße 2c, 2700 Wiener Neustadt, Austria; 2 Bundesanstalt für Materialforschung und-Prüfung (BAM), Unter den Eichen 44–46, 12203 Berlin, Germany

**Keywords:** polymer seals, soft polyurethane, friction, wear, modelling, simulation input data, tribology, digital twin, FEM, material upscaling

## Abstract

Soft polymers such as the investigated polyurethane, characterized by low Young’s moduli and prone to high shear deflection, are frequently applied in pneumatic cylinders. Their performance and lifetime without external lubrication are highly determined by the friction between seal and shaft and the wear rate. FEM simulation has established itself as a tool in seal design processes but requires input values for friction and wear depending on material, load, and velocity. This paper presents a tribological test configuration for long stroke, reciprocating movement, allowing the generation of data which meet the requirements of input parameters for FEM simulations without the geometrical influences of specific seal profiles. A numerical parameter study, performed with an FEM model, revealed the most eligible sample geometry as a flat, disc-shaped sample of the polymer glued on a stiff sample holder. At the same time, the study illustrates that the sensitivity of the contact pressure distribution to Poisson’s ratio and CoF can be minimized by the developed and verified setup. It ensures robust, reliable, and repeatable experimental results with uniform contact pressures and constant contact areas to be used in databases and FEM simulations of seals, enabling upscaling from generically shaped samples to complex seal profiles.

## 1. Introduction

Polymers are widely used in tribological applications such as seals due to their exceptional mechanical and tribological properties, chemical resistance, and cost efficiency and their demand is continuously increasing. Pneumatic cylinders commonly work with so-called soft polymers, a group of polymers with low elastic or viscoelastic properties; examples are rubbers and polyurethane. For optimized systems, in order to streamline the design process for engineers and identify best choice of polymer compound, the finite element method (FEM) is the most efficient procedure today [1]. For these design processes, the material has to be tested, and its material properties characterized and appropriately modelled. While the mechanical characterization and modelling process for materials is well understood and often standardized, the tribological characterization process is often based on comparing friction and wear values between different materials under the same test conditions [2,3]. As a result, geometrical and material influences cannot be separated.

There is a lack of standardization in tribological tests in general and for soft polymers in particular because there is a high variance of test results under seemingly identical conditions in tribological tests [4]. This could be observed for a slight modification of the fixation of the polymer sample, while keeping the same geometry in a pin-on-plate configuration [5]. Comparing the number of constitutive mechanical material models to the number of models accounting for the tribological behavior of the system provided in commercial FEM programs (more than 60 mechanical constitutive models in ANSYS^®^) also reveals the gap between the states of the art of both disciplines. The tribological models only consist of the Coulomb friction model and the Archard wear model, lacking specific parameters for individual materials [6].

Various methods of characterizing the tribological properties of a system of polymers against a metal counter surface can be found in the literature. Laboratory tests with point contacts include the crossed-cylinder contact configuration [7,8], and line contacts can also be archived with a cylinder-on-plate configuration [9,10] or a flat-on-flat contact with a pin- or pad-on-plate/disc configuration under reciprocal [11] or unidirectional [12,13] movement. Those tests to characterize the tribological behavior of a system will here be referred to as model tests. A widespread method to test polymers is the component test, where whole seals are characterized [14,15,16]. The simplified implication that the coefficient of friction (CoF) and wear coefficient follow Amontons’ Law [17], and are thus independent of load and velocity, does not apply for polymers [11,13]. Therefore, the contact pressure distribution plays an important role for the outcome of the measured CoF and wear coefficient in tribological experiments. Additionally, testing soft polymers is especially challenging as high distortions of the sample are highly likely and can cause stick-slip effects [10] which lead to strong hysteresis effects. This results in a strong variance of the measured data and also in a high sensitivity regarding the evaluation method applied in post processing [7].

In this work, the different methods to characterize soft polymers tribologically are assessed via numerical simulations. This basis allows us to understand key influence parameters on the tribological contact and optimize the test setup in order to obtain material specific friction and wear coefficients for soft polymers, such as thermoplastic polyurethane sliding against a steel counter surface under technically dry conditions.

The polymer investigated is tested in a flat-on-flat configuration to ensure a constant contact pressure that is independent of wear occurring during the test over a wide range of applied normal pressures and sliding velocities. Experimental data on friction and wear rate can only be used for prediction of the efficiency and lifetime of materials on the component level if tribological test procedures are carefully chosen. It is essential to standardize all test conditions and parameters, and meticulously record all metadata, such as counter body topography. The values obtained from the experimental test have to be independent of the specimen geometry in order for them to be usable in the upscaling of the material performance in a numerical model. Upscaling refers to the process of simulating a component (a seal, made of the material in question) embedded in a system, such as a pneumatic cylinder, based on input values obtained in a short-time, simple tribological test configuration, here referred to as model test; then, both CoF and wear rate are used as material specific parameters within an FEM simulation that characterizes the tribological interaction of the system for given surface topographies and environmental conditions (humidity, atmospheric pressure etc.).

The investigations of local effects such as stick-slip and surface topography are not included, since the finite element simulation will determine the frictional and wear system behavior based on macroscopic frictional and wear models. The surface topography will be measured and saved as metadata [18], since the surface topography has an influence on the tribological behavior of polymers [19,20]. The setup is designed to minimize the effects of stick-slip originating from compliance of the test setup. Furthermore, the experimental data to be used as input for FEM simulations should be independent of the deformation of the soft polymer sample.

## 2. Materials and Methods

### 2.1. Materials

The material investigated in this study is a thermoplastic polyurethane with the commercial name HPU premium supplied by Trygonal Group GmbH [21]. This polymer is used in dynamic seal applications under reciprocal, linear movement, with typical strokes of several centimeters, and in dry conditions. In order to predict the behavior of the polymer component in a sealing system using FEM simulations, the mechanical and the tribological material properties must be tested and modelled.

Typically, thermoplastic polyurethane is modelled with hyperelastic material models [22,23]. For this simulation setup the neo-Hookean model is used, which represents the mechanical response of TPU in terms of the stress–strain curve of a uniaxial tensile test, accurate up to a strain of 100% [24]. The material formulation of hyperplastic material is based on the strain energy density function, which is characterized for the neo-Hookean model with two material parameters. In ANSYS, the strain energy density function W is formulated using the initial shear modulus G and the incompressible parameter $D_{1}$ [25].

(1) $$W=\frac{G}{2}\;\left({\;\bar{I}}_{1}-3\right)+\frac{1}{D_{1}}{\left(J-1\right)}^{2}$$

Here, ${\;\bar{I}}_{1}$ is the deviatoric strain invariant and J is the determinant of the deformation gradient F. For convenience, the initial shear modulus G and the incompressibility parameter $D_{1}$ are replaced with the initial tangent Young’s modulus E (from here on referred to as Young’s modulus) and the Poisson’s ratio ν. The relationship between these parameters is given in Equation (2). For isotropic materials, the Poisson’s ratios vary between −1 and 0.5 as the corresponding elastic moduli, e.g., Young’s modulus, shear modulus, and the compression modulus, must be positive. As auxetic materials [26,27] (ν<0) with characteristically cellular structures do not provide adequate properties for tribological applications with high contact pressures, the investigated materials are limited here to Poisson’s ratios between 0 and 0.5.

(2) $$G=\frac{E}{2\left(1+\nu \right)};{\;D}_{1}=\frac{2}{K}=\frac{6\left(1-2\nu \right)}{E}$$

where K is the bulk modulus.

### 2.2. Methods

Tribological testing conditions are especially challenging for soft polymers operating in reciprocating motion as high material deflections result in non-uniform contact pressures with the nominal contact area; both of which change at each turning point. This chapter describes the development of a model test to ensure a testing method capable of generating frictional and wear data to directly formulate models usable in FEM simulations. A model test is characterized by its simple sample configuration and comparably short testing times. It has the aim to eliminate geometrical effects, e.g., from seal profiles, on the results and enable standardized testing procedures.

#### 2.2.1. Designing Experimental Tests for Soft Polymers

While the stresses in a uniaxial tensile test are constant over the cross-sectional area of the specimen $A_{0}$, this is usually not the case for the contact pressure in a tribological test with soft polymers due to the deformation of the specimen caused by the frictional shear stresses. Consequently, the measured values do describe the system behavior, but not necessary the contact interactions on a local level which are the data of interest for the derivation of friction and wear models.

The output values of a tribological test usually are given by:

- the normal load $F_{N}$;
- the frictional force $F_{\mathrm{fric}}$;
- the relative velocity $v_{\mathrm{rel}}$ between sample and counter body;
- the loss of volume due to wear $V_{w}$ usually determined by the mass loss assuming constant material density.

**Figure 1:**
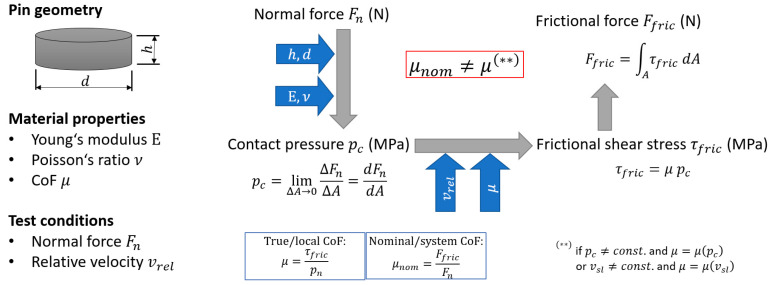
Comparison between nominal and true coefficient of friction for arbitrary contact pressure distributions and materials with pressure und velocity dependent frictional behavior.

A tribological test setup needs to be found that provides the ability to derive directly the local CoF and the specific wear rate with the measured values given. This is especially critical for soft polymers as thermoplastic polyurethanes show a strong dependence on the measured CoF and the contact pressure. Figure 1 illustrates the relationship between the nominal CoF measured and the true CoF in the contact. Here, it is shown that the true CoF can only be derived directly from the nominal CoF if the contact pressure is constant over the contact surface, or if the CoF is constant and independent of the contact pressure. Both criteria are usually not fulfilled and deriving the local CoF based on the system output results becomes unfeasible. Additionally, stick-slip effects cause a difference between the relative movement given and the actual sliding velocity and alter the frictional forces as polymers typically show velocity dependent frictional behavior [28].

Direct determination of the CoF from the frictional forces is only possible for the test setups that ensure a nearly constant contact pressure over the contact area. Additionally, the stresses at the edges should be minimal to guarantee reliable test conditions. Only then is the CoF constant over most of the contact area and is determinable from the measured forces. Naturally, soft polymers are very prone to exhibit stick-slip effects during reciprocating sliding experiments. Due to the high deformability in the elastic regime, full cylinders usually show pronounced stick-slip even over strokes of several centimeters. Therefore, deriving a CoF for sliding as a basis for simulations, such as full-scale seal FEM models of the whole pneumatic cylinder system, is utterly impossible.

The test setup should provide a high stiffness to minimize stick-slip effects that can lead to a difference in the relative velocity between contact body of the polymer sample and the counter body and the actual sliding velocity in the contact. Finally, the contact area should not change with wear. The volume loss due to wear is determined over the difference in mass of the sample before and after testing. Consequently, the specific wear rate is determined in post-processing based on the normal force given and the total sliding distance for a given contact pressure and a relative velocity. Inconsistent test conditions, e.g., a reduction of the contact pressure over time due to increasing contact area caused by wear renders the determination of the correct wear behavior very difficult. The wear coefficient includes all surface effects, such as plastic deformation as well as the removal of wear particles. Therefore, the plastic deformation is not included separately in the FEM simulation.

**Figure 2:**
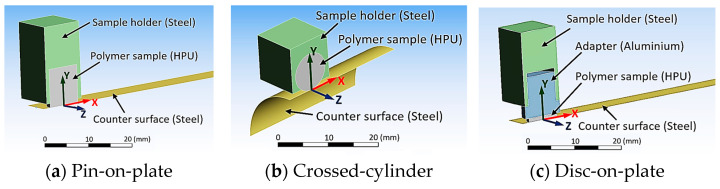
Experimental setup to characterize the tribological behavior of soft polymers with (**a**) polymer pin-on-plate, (**b**) crossed-cylinder configuration and (**c**) a thin polymer disc-on-plate configuration.

To evaluate possible test setups, three configurations were numerically investigated in a pre-study (see Figure 2). All configurations were performed with the same material model formulations. For steel (E =200 GPa, ν =0.3) and aluminum (E =71 GPa, ν =0.33), linear material models were used, and the hyperelastic neo-Hookean model was used for HPU (E =10 MPa, ν =0.4). The CoF was set to be μ =0.5 and the geometrical parameters of the polymer sample (the diameter d =8 mm and the cantilever length of the pin h =1 mm) were also the same for all test configurations. The normal load was chosen such that the average contact pressure results in 1 MPa, which is a typical average pressure occurring in seal contacts (50 N for pin-on-plate and disc on-plate configuration and 4 N for crossed-cylinder-configuration). The counter surfaces were considered as rigid bodies, with a radius r =12.5 mm for the crossed-cylinder configuration. The counter surfaces also performed a relative movement in negative x-direction to generate a frictional sliding contact between the polymer and the counter surface. To reduce computational efforts, the symmetry plane in every model was used and only half of the model was computed.

Additionally, the contact between the lateral surface of the polymer pin and steel holder in the pin-on-plate configuration (see Figure 2a) was considered as bonded and sliding contact to consider the effect of slipping between pin and sample holder. This effect surely depends on the CoF between the sample holder and the polymer sample as well as the clamping force, the normal load, and the material stiffnesses. Here, the extreme cases with an infinitely high CoF (bonded) and a CoF of zero (sliding) are considered. 

**Figure 3:**
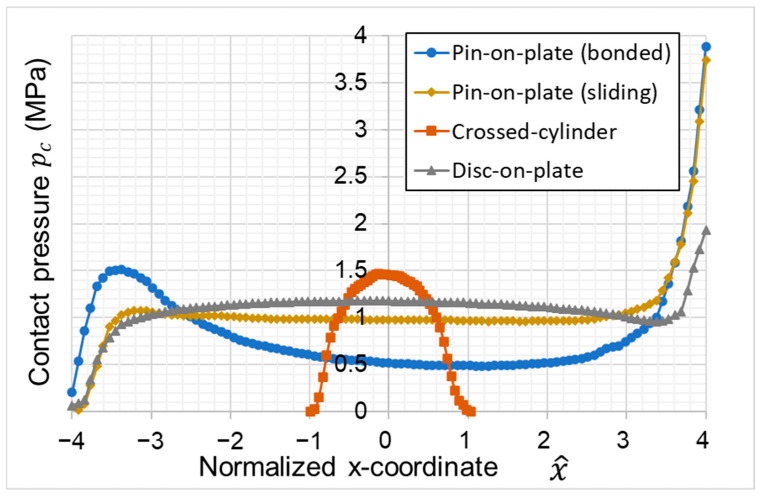
Comparison between contact pressure distributions for different test setup configurations.

The comparison of the contact pressure in sliding direction (*x*-axis) between the four test cases shows clear differences (see Figure 3). The pin-on-plate configuration shows the highest contact pressures at the leading edge of the polymer pin. While the contact pressure is nearly constant over a large area of the surface for a sliding contact between the polymer pin and the holder, a bonded contact, e.g., due to a higher clamping force from the sample holder which leads to increased contact pressure on the trailing edge and a significant reduction in contact pressure in the center of the pin. For both configurations, the contact pressure at the leading edge is the same. The crossed-cylinder configuration shows the maximum contact pressure at the center of the contact area. The contact pressure distribution is nearly the same at the leading edge and trailing edge. The disc-on-plate configuration shows a similar contact pressure distribution as the pin-on-plate configuration, but the contact pressure at the leading edge is significantly reduced.

Based on these results, the disc-on-plate is further investigated as it shows a nearly uniform contact pressure over a large area of the contact surface and the maximum contact pressure at the leading edge could be reduced significantly compared to the pin-on-plate configuration. To further improve the contact pressure distribution, the following influence parameters have been studied:

- Height of polymer disc h;
- Diameter of polymer disc d;
- Nominal contact pressure $p_{n}$, given by normal load $F_{N}$ and the cross-sectional area $A_{0}$: $p_{n}=\frac{F_{n}}{A_{0}}$;
- Young’s modulus E;
- Poisson’s ratio ν;
- CoF μ.

**Figure 4:**
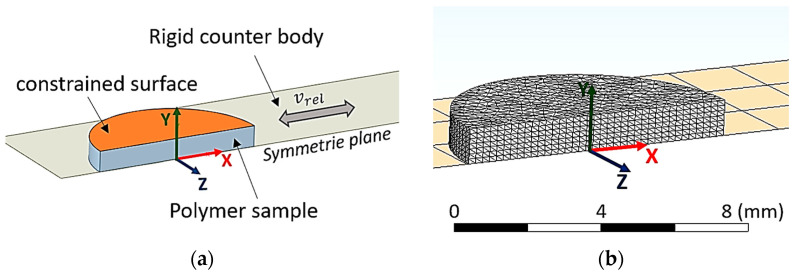
Simulation setup for parameter study with 14,115 linear tetrahedral elements: (**a**) boundary conditions; (**b**) mesh of the discretized model.

The numerical design study was performed on a reduced model that only considers the polymer pin; since the Young’s modulus of aluminum ($E_{\mathrm{Al}}=70\;\mathrm{GPa}$) and steel ($E_{\mathrm{St}}$ = 200 GPa) is more than three orders of magnitude higher, those components were simplified to rigid bodies. The applied boundary conditions and the descriptive model of polymer pin are shown in Figure 4. The details of this model are as follows. The nodes on the upper surface of the polymer pin are constrained to a circle where only lateral movement in y direction is allowed; no movement in x and z direction is possible. Additionally, the area of the circle (indicated in orange in Figure 4) is not allowed to twist which is ensured by no relative movement of the nodes in y direction. These boundary conditions resemble the connection of the polymer disc onto the rigid aluminum sample holder. These boundary conditions allowed the application of the normal load on the disc on a single node. After applying the load, the rigid counter body performs a defined relative movement normal to the loading direction. The visible mesh of this body (shown in Figure 4b) represents the contact elements and does not allow the penetration of the polymer disc. Linear tetrahedral elements were used to discretize the polymer sample to increase stability of the simulation which is necessary because of the high local distortions.

#### 2.2.2. Experimental Tribological Model Tests

**Figure 5:**
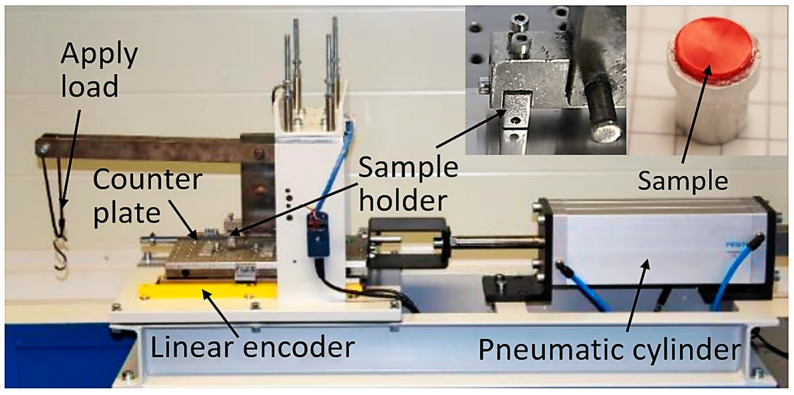
Experimental setup to characterize the tribological behavior of soft polymers: Sample holder and polymer sample (upper right); Experimental test stand at AC2T research GmbH for reciprocal tribological experiments.

Based on the findings of the design study, the geometry of the model test sample to characterize the HPU polymer against a lapped 100Cr6 steel plate was defined. Figure 5 shows the new model test technique the authors present in this paper: a polymer disc glued to an aluminum adapter. This setup aims at a minimization of deflection to such a degree that contact pressures are uniform over as large as possible an amount of the nominal contact area and, most importantly, this pressure remains independent of the polymer wear. Any other sample shape such as full polymer pin or crossed-cylinder exhibits massive changes of the contact pressure throughout the experiment. Consequently, their results prove inappropriate in providing CoF and wear rate data to parameterize various polymer compounds for FEM simulations. A temperature sensor was attached to the adaptor, onto which the polymer pin was glued; however, due to the excellent insulation properties of the polymer, the actual temperature in the contact could not be detected. Additionally, any heat created in the area of contact is assumed to be dissipated by the thermal conductivity of a high mass steel counter body. Therefore, the temperature is considered to be moderate throughout the test; a simulation of the contact temperature was not implemented in the present work.

The CoF of the tribological system tested is determined over the constant velocity region of the test, which ranges from 20% to 80% displacement of the stroke, respectively, from position −20 to position 20 mm for a stroke of 50 mm for the forward and backward stroke in order to obtain one value per cycle. Additionally, the maximum value of the frictional force is determined for each stroke to determine the static CoF of the system. The CoF over cycles and the maximum CoF are then used to determine the average value over those cycles in the steady-state regime. The standard deviation of these values results from the three repetitions of each test.

The evaluated CoF corresponding to a certain contact pressure and sliding velocity are subsequently used to derive an appropriate friction model capable of describing the frictional behavior of the HPU polymer as a function of the contact pressure and the sliding velocity.

#### 2.2.3. Verification of Model Test

**Figure 6:**
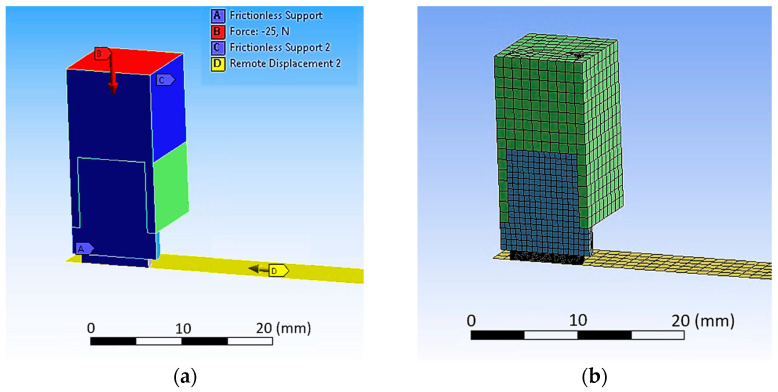
Full 3D model of the test setup including the sample holder and the adapter with (**a**) applied boundary conditions and (**b**) numerical mesh with 39,526 elements.

To ensure the generation of trusted data to derive friction and wear models from the model test developed, the derived friction model was implemented in an FEM simulation reproducing one test cycle of a model test. Comparing the nominal CoF of the simulation to the CoF measured in the model test ensures the accuracy of the derived friction model based on the experimental data; thus, it proves the trusted workflow of the tribological characterization of soft polymers. Figure 6 shows the numerical model with the boundary conditions applied and the numerical mesh.

## 3. Results

This chapter presents the optimized geometry found based on the results of the design study, the experimentally derived CoF for different loading scenarios, the derived frictional model, and the verification of the frictional model, proving the procedure capable of producing trusted data using this newly developed testing method.

### 3.1. Design Study and Derived Empirical Relationships

The values of the parameters for the design study are shown in Table 1. The values in bold indicate the set of parameters that is left constant while sweeping each of the parameters.

The parameter study of the six influence parameters resulted in a total of twenty-two simulations. The main outputs of interest are the contact pressure distribution and the influence of the model parameters in the design of a polymer disc with minimal edge effects due to deformation caused by the frictional forces.

The results of the numerical design parameter study are presented in dimensionless values in order to allow a direct comparison between the parameter variations. The dimensionless values are defined in Table 2.

**Figure 7:**
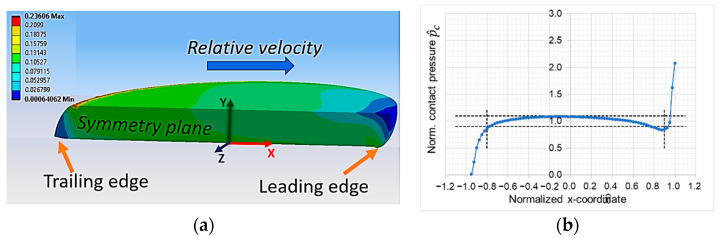
(**a**) Equivalent strain of deformed polymer pin (deformation is scaled by a factor of three); (**b**) the corresponding normalized contact pressure along the symmetry axis for base parameters.

Figure 7a shows the deformation of the polymer pin for the base conditions (see values presented in Table 1 in bold). The elastic deformation due to the frictional forces is already clearly visible for a scaling factor of three. The resulting contact pressure distribution along the x-coordinate during sliding is presented in Figure 7b. Here, it shows that the frictional forces in the contact cause an unsymmetric contact pressure distribution. The maximum contact pressure is at the leading edge of the polymer pin. Here, most of the wear occurs, which was also observed in previous experimental tests in pin-on-plate configuration. The contact pressure in the trailing edge, however, decreases; lifting of the polymer pin can occur, which results in a contact pressure of zero at the trailing edge (see contact pressure in Figure 7b). In order to prevent severe wear at the leading edges and a radical drop of contact pressure, the effects of non-uniform contact pressure at the edges should be minimized. To qualify the contact pressure distribution at certain test conditions, limits for the acceptable range of the contact pressures were defined and are shown in vertical dashed lines in Figure 7b. The horizontal dashed lines present the limits of the contact pressure in range of ±10% of the nominal contact pressure and the vertical lines enclose a contact area of ~90% of the nominal contact area. A contact pressure distribution in the described pressure limits within the vertical limits of the contact area is assumed to be acceptable and leads to stable experimental test conditions. Stable conditions are characterized by little scatter of the friction force along a hysteresis curve and little variations between different hysteresis cycles over time once the run-in period is finished.

**Figure 8:**
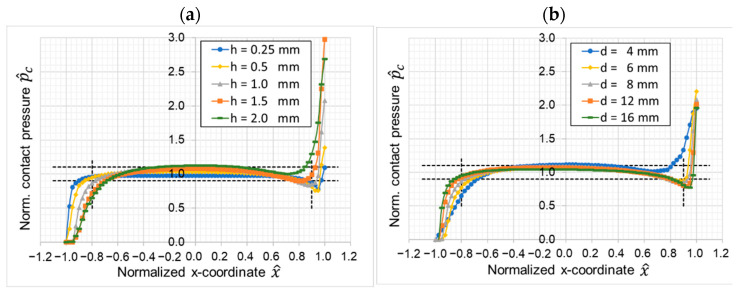
Variation of sample geometry (**a**) variation of height; (**b**) variation of diameter.

The first parameter variation investigates the geometrical influence of the polymer sample in terms of diameter and height of the pin. Figure 8 shows the normalized contact pressure for (a) different sample heights and (b) different sample diameters. The variations of diameter and height show both significant influence on the pressure distribution at the leading edge and the trailing edge. The contact pressure in the center of the pin shows only slight changes. An improved contact pressure distribution can be observed for reduced pin heights and increased pin diameters. The effects on the edges can be reduced and the contact pressure in the center decreased slightly to generate a more even contact pressure distribution. The choice of the sample geometry is demonstrated to have a significant influence on the contact pressure distribution and the stability of the performed tests.

**Figure 9:**
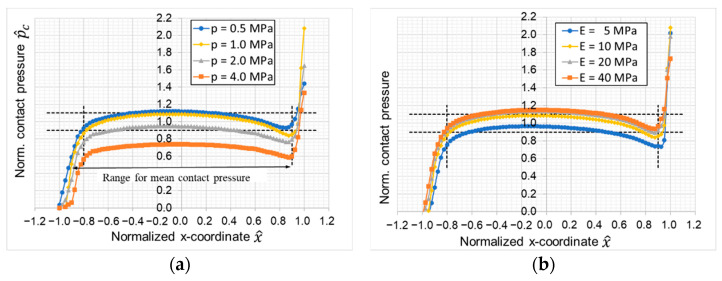
Influence of (**a**) nominal load and (**b**) Young’s modulus of the polymer.

Next, the influence of the sample load in terms of the nominal contact pressure and the polymeric material stiffness expressed in the Young’s modulus were investigated. Here, it can be observed that for both parameter variations the profile of the contact pressure shows no significant change (see Figure 9). However, for higher loads, the level of the normalized contact pressure decreases because of increased deformations of the polymer. Likewise, lower Young’s moduli lead to higher lateral deformations and consequently to increased contact areas and lower contact pressures. Nevertheless, the influence of these two parameters on the test conditions of the test appear to be rather small.

**Figure 10:**
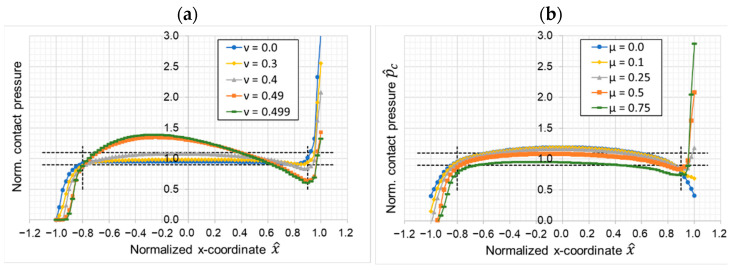
Influence of (**a**) Poisson’s ratio and (**b**) coefficient of friction.

The last two parameter variations investigate the non-dimensional material parameters Poisson’s ratio and the tribological system parameter CoF. Both parameters show a strong effect on the pressure distribution at the leading edge. Materials with a high Poisson’s ratio (ν>0.4) show a more curved contact pressure distribution with a maximum contact pressure in the center of the sample. Here, the contact pressure falls to zero at the trailing edge, which indicates a lifting of the edge. Conversely, a high CoF mainly effects the leading edge. While a CoF of zero results in a perfectly symmetric contact pressure distribution and the contact pressure at the leading edge is the same as on the trailing edge, a higher CoF reduces the contact pressure at the trailing edge and significantly increases the contact pressure at the leading edge. Figure 10b shows that a high CoF can cause contact pressures at the leading edge that is a multiple of the nominal contact pressure. The pressure in the center of the pin reduces slightly with high CoF. A high Poisson’s ratio as well as a high CoF seem to be critical for stable test conditions since both lead to a more uneven contact pressure distribution over the contact surface. Both properties are typically high for polymeric materials.

The investigated study shows similar influences on the contact pressure distribution for varying the polymer disc height and diameter. A similar effect could also be observed for varying nominal contact pressure and the Young’s modulus. The influence of the Poisson’s ratio and the CoF on the contact pressure distribution are more complex and effect the contact pressure distribution differently.

**Figure 11:**
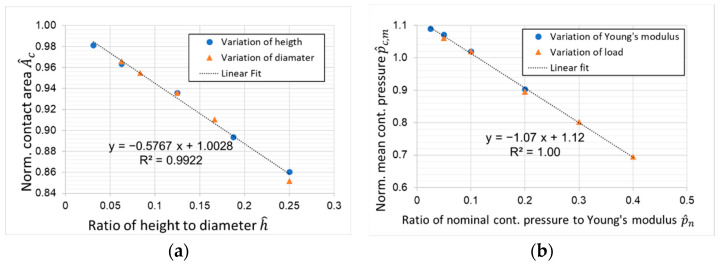
(**a**) Normalized contact area with contact pressure within ±10% of nominal pressure for different specimen geometries; (**b**) normalized mean contact.

To evaluate the contact pressure distribution, the area of the contacting surface with a contact pressure in range ±10% of the nominal contact pressure is computed and compared to the nominal contact area. For different height to diameter ratios, here, a linear relationship could be found (see Figure 11a). For a minimum 90% of the contact area fulfilling this condition the ratio between height and diameter should be less than 1/6. Note that the linear regression has a limit of 100% as the ratio of height to diameter approaches zero.

The normalized mean contact pressure ${\;\hat{p}}_{c,m}$ is defined by the area within the boundaries that enclose 90% of the nominal area $A_{0}$. Comparing ${\;\hat{p}}_{c,m}$ to the ratio between applied nominal contact pressure $p_{n}$ and Young’s modulus E results in a linear relationship as well (see Figure 11b). This lateral elastic deformation appears not to be relevant for the stability of the experimental test as long as the material is loaded within its elastic limits. However, for high deformations, a change of the contact area has to be considered when deriving the frictional model. It is important to note that the limiting load depends on the yield stress and the CoF, since the elastic limit results from the combined load of normal stress and frictional shear stress [11].

### 3.2. Experimental Results and Friction Model

Based on the observations from the data, only the flat-on-flat contacts guarantee a uniform and constant contact pressure that is independent of wear. Additionally, gluing the soft polymer onto a much stiffer material (e.g., Aluminum) leads to an additional improvement of the contact pressure distribution. A sample geometry with 8 mm in diameter and 1 mm in height was chosen to characterize the HPU polymer material. According to the relationship derived between geometry and contact area (see Figure 11a), this results in a normalized contact area ${\;\hat{A}}_{c}\;$of 93%. Based on the parameter study presented in this work, the sample geometry can be designed accordingly for future model tests with other soft polymers. It will not be necessary to perform this parameter study anew each time. The test procedure was performed for two different nominal contact pressure loads and two velocities with three repetitions each, resulting in a total of twelve experimental tests. See test matrix in Table 3.

**Figure 12:**
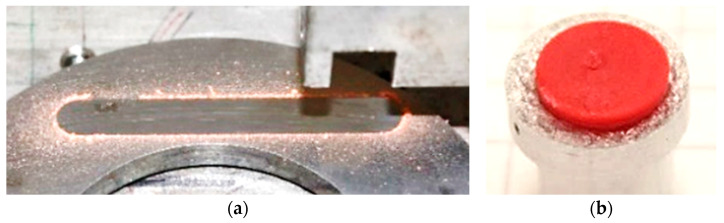
Tribological characterization of HPU premium: (**a**) wear track after testing procedure; (**b**) worn sample after test.

Figure 12 shows the sample holder, counter surface, and polymer sample in more detail after the experimental testing procedure.

**Figure 13:**
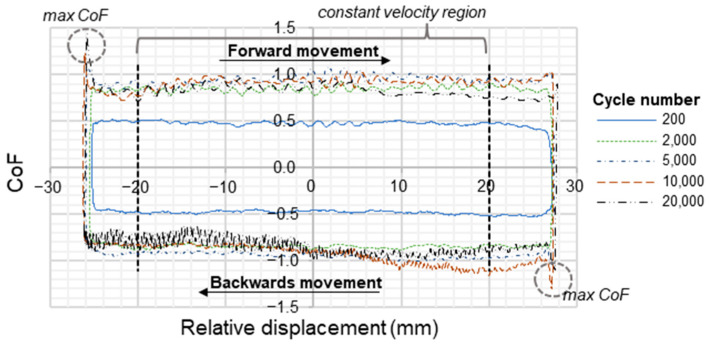
Coefficient of friction resoved over displacement at different cycle numbers for normal pressure of 1 MPa and 100 mm/s for dry friction.

Figure 13 shows a typical evolution of the CoF over one cycle at different points in time during the test. In the case of a uniform contact pressure, the differentiation between a nominal and true CoF (as laid out in Figure 1) is no longer required. The movement in the diagram is clockwise (indicated by the black arrows). During the running-in of the test, the frictional forces are the lowest and increase until reaching stable conditions after 2000 cycles. Here, the specimen also developed a more pronounced velocity dependent behavior, which is indicated by the peaks of the nominal CoF at the turning points. This peak indicates the static CoF of this material in combination with the selected counter body surface. The sliding part, which extends over several centimeters in this test, reveals only minor fluctuations that are clearly outreached by the peak values at the turning points. Thus, fairly constant sliding conditions are obtained in this test.

The CoF of the selected test conditions is determined by averaging the CoF measured from 20% to 80% displacement of the stroke, respectively, from −20 to 20 mm for a stroke of 50 mm. The static CoF is determined using the maximal values of the CoF of each forward and backwards movement and averaging these values over all cycles. All these values are determined within the steady-state condition of the experiment, where stable conditions have been reached (minimal variation of CoF over different hysteresis cycles and minimal scatter of CoF within each hysteresis cycle).

The frictional behavior of the polymer material is modelled with a combined empirical equation, which is able to model the velocity and contact pressure influences on the CoF. The velocity dependence of the CoF $\mu_{v}$ is formulated according to Bellson and Hallquist [29] in Equation (3) with a static CoF $\mu_{s}$, a dynamic coefficient CoF $\mu_{d}$, a constant c and the sliding velocity v:

(3) $$\mu_{v}\left(v\right)=\mu_{d}+\left(\mu_{s}-\mu_{d}\right)e^{-c\left|v\right|}.$$

Hallquist refers to c as the decay constant, which describes—e.g., in classical tensile test experiments—the decrease of the stress within the body after a short time application of a given strain. So, this coefficient quantifies the viscoelastic property of a polymer because the internally stored energy dissipates due to viscoelastic material properties. A sliding test closely resembles a compression test combined with a sliding movement. If the relative movement is too fast, then the asperities indent the polymer material not as deeply because the material behaves stiffer for high strain rates.

The accumulated strain rate, which also considers the incremental energy dissipation, can be equalized with the coefficient c according to Hallquist. Here, the strain rate is equalized with the sliding velocity. Consequently, the sliding velocity v, which reaches a certain critical velocity $v_{0}$, can describe that limit above which the material behaves more stiffly. Above this sliding velocity, the counter body asperities can no longer indent the polymer as deeply as they do for slow sliding velocities. As a result, Equation (3) can be written as:

(4) $$\mu \left(v\right)=\mu_{d}+\left(\mu_{s}-\mu_{d}\right)\exp\left(-\frac{v}{v_{0}}\right).$$

The current study focuses on friction and dissipation effects acting when the polymer slides over the asperities. Naturally, the degree of indentation changes with the contact pressure $p_{c}$ applied. The effects on the CoF in an elastic contact due to higher loads, causing increased asperity indentation and an increased real contact area, are described by an empirical relationship [28]:

(5) $$\mu \left(p_{c}\;\right)={C\;p}_{c}^{-n}.$$

Here, C describes a constant for thermally controlled conditions and constant sliding speeds. Based on this finding, the contact pressure dependence of the CoF can be described with a power law with a reference contact pressure $p_{0}$ and an exponent n. Provided the asperities can be described by a perfect ball indenting elastically, the maximal contact pressure $p_{0}$ could be described by an exponent n = 1/3. However, the literature values for n diverge. For example, Sinha et al. [28] found values of n = 0.26 by fitting experimental data obtained from sliding POM-H at different pressures in elastic regimes. They found values for n >> 1/3 in viscoelastic regimes at extremely low sliding velocities.

In order to provide an equation describing a pressure and velocity dependance, the current study combined Equations (4) and (5). As the coefficient C in Equation (5) is to be interpreted as a coefficient of friction, we implement the velocity dependency of Equation (4) instead of C. This approach forms the new Equation (6). With the assumption that n does not change with different sliding velocities this empirical equation can describe the CoF as a function of contact pressure $p_{c}$ and sliding velocity $v_{0}$.

(6) $$\mu \left(p_{c},v\right)=\left[\mu_{d}+\left(\mu_{s}-\mu_{d}\right)\;\exp\left(-\frac{v}{v_{0}}\right)\right]{\left(\frac{p_{c}}{p_{0}}\right)}^{-n}$$

Note, here, that the CoF approaches infinity as the contact pressure approaches zero; the resulting frictional stresses still become zero with n<1. The model fit is performed for eight experimental data points. These are given by the four mean frictional values representing the CoF for the given test conditions and the four maximum values of the model tests representing the static CoF at the turning points of the reciprocal movement. The material parameter determined for HPU premium from Trygonal can be found in Table 4. The pressure exponent n was found to give the best contact pressure dependent relationship for a value of 0.26. This value for n is also found for other polymers with an elastic contact behavior [26].

The empirical friction and wear models used here do not explicitly take roughness into account; therefore, roughness is included through the model parameter. These will change with different surface topographies for both the friction and the wear model. On a laboratory scale, the running-in of soft polymers is considered not to influence the tribological behavior in the steady state of a test significantly. Therefore, the running-in is not considered in the models applied.

For polymers with no viscoelastic properties and contact surface effects, e.g., adhesion, the static CoF and the dynamic CoF equal each other. In Equation (6), the exponential relationship with the sliding velocity vanishes and the CoF is henceforth only dependent on the contact pressure.

**Figure 14:**
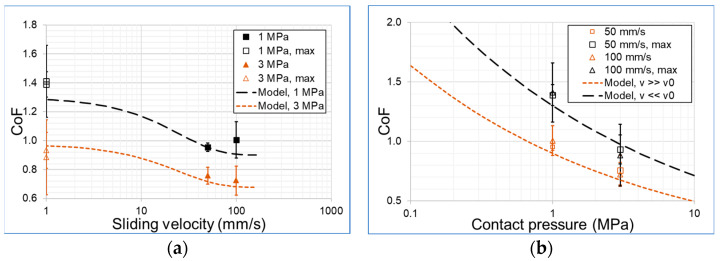
Model fit for frictional model for (**a**) sliding velocity and (**b**) contact pressure vs coefficient of friction.

Figure 14 shows the CoF measured, including the standard deviation and the frictional model with the parameters given in Table 4 versus (a) the sliding velocity and (b) the contact pressure. The results show that the static/maximal CoF for all model tests performed are within the standard deviation for the different sliding velocities. A differentiation between the maximal CoF is thus only necessary for different contact pressures. The coefficient of friction model describes the frictional behavior of the polymer in good agreement within the standard deviation of the experimental data. It could be easily parameterized for other polymer compounds with the support of the respective model test data.

### 3.3. Verification

**Figure 15:**
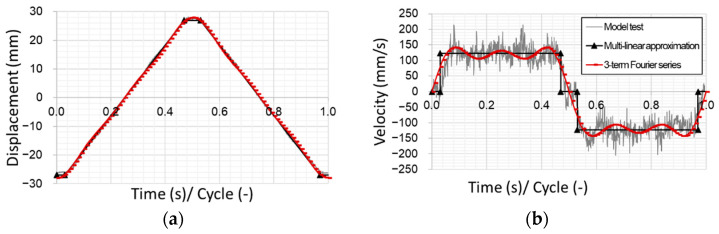
(**a**) Displacement and (**b**) velocity over time and for experimental test and simulation.

To verify the derived frictional model against experimental data, cycle 16,200 of a model test with a nominal pressure of 1 MPa and a sliding velocity of 100 mm/s was selected as representative for the experimental tests. This test was then replicated by a numerical FEM setup and the resulting nominal CoF of the simulation and the experiment were compared to provide some insight into the accuracy of the frictional model and the testing procedure. For a proper representation of the reciprocal movement of the counter surface, the data points measured by the linear encoder during the experimental test were used to choose a suitable approach. A multilinear fit as well as the first three terms of the Fourier series were used to replicate the displacement (see Figure 15a). Both approaches are good fits for the displacement over time, but the multilinear fit shows discontinuity at the turning points plotting the velocity over time (see Figure 15b). The Fourier series shows a continuous behavior here and represents the velocity domain much better.

The data input table for the multilinear fit can be found in Table 5.

The first three terms of the Fourier series for triangular wave function are given by:

(7) $$s\left(t\right)=-\frac{8h}{\pi^{2}}\overset{N}{\underset{k=1}{\sum }}\frac{\cos2\pi \left(2k-1\right)t}{{\left(2k-1\right)}^{2}}\;\mathrm{with}\;h\;=30\;\mathrm{mm},\;N=3.$$

**Figure 16:**
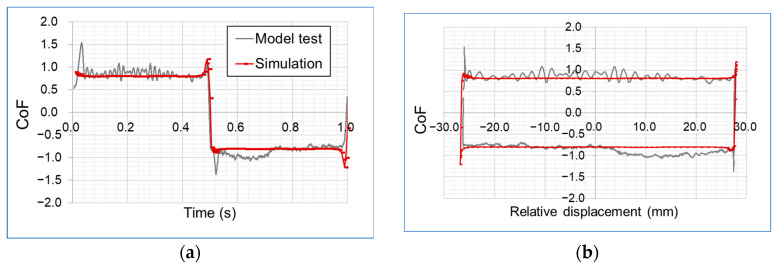
Coefficient of friction over (**a**) time and (**b**) relative displacement.

Comparing the results of the nominal CoF over time and displacement for the experimental test, the simulation shows good agreement with respect to the fluctuations of the signals of the experiments measured (see Figure 16). The slight velocity changes during the stroke seem to have no influence on the velocity dependence. At the turning points, both the simulation and the experiment reach the maximum CoF. While the experiment reaches this maximum at the beginning of the stroke, the simulation shows the maximum in the end of the stroke. This effect is likely caused by the change in velocity at the turning for the experiment, visible in Figure 15b.

The agreement between the experimental data of the model test and the simulation proves that the testing conditions provide trusted data from which to directly derive a frictional model as a function of contact pressure and sliding velocity useful for finite element simulations to evaluate the tribological performance of a system.

## 4. Discussion

This new approach of characterizing soft polymers by gluing polymer discs with optimized diameter to thickness ratios on a much harder substrate shows a significant improved contact pressure distribution. This way, it is possible to use a flat-on-flat configuration to characterize soft polymers and use the generated data to directly derive tribological models useful for FEM simulations.

### 4.1. Numerical Design Study and Verification of the Frictional Model

The pre-study investigating of a full pin flat-on-flat configuration and a crossed-cylinder configuration shows that a flat-on-flat configuration can provide a constant contact pressure over a large area of the contact, which makes it possible to directly retrieve a CoF for a given nominal contact pressure. The full-cylinder configuration displays the maximum contact pressure at the leading edge, which is a multiple of the nominal contact pressure and could lead to severe wear during tests and instable test conditions (see Figure 3). The crossed-cylinder configuration, on the other hand, shows the maximum contact pressure in the center of the sample and no critical conditions at the leading edge, which indicates much more stable test conditions. However, the contact area and contact pressure is highly dependent on the sample geometry, the mechanical material properties, and the occurring wear during the test; this makes it difficult to refer the measured CoF to a specific contact pressure. The here-developed polymer disc glued onto much a harder substrate combines the advantages of both test setups by providing a nearly constant contact pressure over most of the contact area that is nearly independent of the mechanical properties of the soft polymer and the occurring wear, while simultaneously showing a significantly reduced contact pressure at the leading edge, thus providing highly improved test conditions.

While the geometrical dimensions of the sample can be altered for an improved test procedure, parameters such as the mechanical material properties are given by the selected compound materials and required pressure ranges are determined by the application. The tribological properties are inherently the result of all latter parameters as well as of the choice of the counter body surface and type of lubrication. As the current work is dedicated to dry contacts between soft polymers against much harder counter surfaces, no external lubrication is applied in the model tests nor simulated explicitly in the FEM model. These types of polymers are designed to operate in pneumatic actuators operating with air or other gases such as nitrogen. A parameter study investigating the various model-influencing parameters on the tribological test setup shows a highly non-uniform contact pressure distribution between the polymer test samples and the counter body for high CoFs and Poisson’s ratios above 0.4. Both properties are typically found in soft polymers, e.g., TPU, with a Poisson’s ratio ranging from 0.48 to 0.5 [30,31], which is presumably the main reason stable and reproducible tests are hard to perform. On the other hand, it was found that a low ratio between height and diameter of the polymer disc improved the contact pressure distribution. The choice of a reasonably low sample height for a cylindrical sample with a given diameter can alter an instable test of a full-cylinder flat-on-flat configuration into a stable tribological experiment.

A linear relationship was found between the sample geometry parameters, height and diameter, and contact pressure distribution (see Figure 11a). A constant contact pressure over 90% of the contact area leads to stable operation during the test. In order to achieve those uniform contact pressure conditions, the ratio between height and diameter has to be below 1/6. The material stiffness was found to have no significant influence on the contact pressure distribution; therefore, the linear relationship between disc dimensions and contact pressure distribution will apply for any other soft polymer as well.

A second linear relationship was found that describes the reduction of the average contact pressure due to the elastic deformation of the pin. For very high loads, the contact pressure based on the initial cross-sectional area of the pin should be considered to derive the correct relationship between pressure and the corresponding tribological properties.

### 4.2. Standardization of Tests for Upscaling Material Performance

The presented workflow for testing soft polymers demonstrates new guidelines for a sample design for a flat-on-flat contact against much harder materials, designed to ensure a nearly constant contact pressure over the contacting surface that shows little influence of the material properties and wear throughout the experimental tribological test. Providing all necessary metadata of the test generates highly reproducible test data to ensure comparability between test results, while also providing data that can be used as input for numerical simulations.

A common procedure of testing a normed standard material under the same test conditions is to express the tribological properties relative to this standard material in order to compare the material performance of a different polymer on a different experimental setup. With the better understanding of the influence parameters presented here in this work, this additional testing of the standard material will arguably be obsolete, leading to a much faster testing process. This procedure was, among other things, developed within the testbed project i-Tribomat (i-tribomat.eu, Grant agreement No. 814494) to follow the FAIR (Findability, Accessibility, Interoperability and Reusability) principles [32] and generate a database of friction and wear data of tested polymers, whose schema was designed by ANSYS Granta with the partners. This database is used to store the tribological material data including all meta data for each experiment, e.g., the testing conditions, testing device, date, and 3D topography of the counter. Data will be accessible commercially via the ETC.

## 5. Conclusions

A well-known challenge in tribology is that little changes in the test setup can alter the result of the testing procedure completely. In particular, reciprocal tests with soft polymers sliding against hard counter surfaces result in high distortions caused by high coefficients of friction and the experiment can descend into unstable conditions quickly. Typically, the choice for such tests falls to point or line contact realized with a ball-on-plate, cylinder-on-plate, or crossed-cylinder configuration. These are often appreciated for their comparably easy setup. However, the contact area and contact pressure of these tests are highly dependent on the mechanical properties of the polymers. The used sample geometries, the load, and the occurring wear, which increases the contact surface, lowers the contact pressure and usually results in a change of the CoF and wear rate.

The only way to perform a test that does not change its contact conditions is a flat-on-flat configuration. Numerical investigations of different setups such as the pin-on-plate and crossed-cylinder configuration showed significant differences in the contact pressure over the tribological contact area during sliding. The pin-on-plate configuration shows a highly asymmetric contact pressure distribution across the contact area with contact pressures significantly increased at the leading edge. The crossed-cylinder configuration leads to a nearly symmetric contact pressure distribution and much lower maximal contact pressure in the center of the polymer sample compared to, e.g., the pin-on-plate setup. For robust test conditions, the maximal contact pressure should not be significantly higher than the nominal contact pressure to prevent uneven wear in the contact area. A new developed setup with a thin polymer disc, glued onto a specifically designed aluminum adapter, shows a significant reduction of the contact pressure at the leading edge compared to the pin-on-disc test. A numerical parameter study was performed to investigate the influences of the polymer disc parameters on the contact pressure distribution. The study has shown that:

- A polymer disc with a height to diameter ratio of less than 1/6 was demonstrated to be most eligible for robust test procedures and uniform contact pressures. This ensures that occurring wear does not change contact pressure;
- The Young’s modulus, which is typically low for soft polyurethane, has little influence on the contact pressure distribution of the newly developed disc-on-plate configuration as long as the ratio of the nominal contact pressure and Young´s modus is below 0.1;
- Poisson’s ratios of the polymer above 0.4 significantly reduce the uniformity of the contact pressure distribution and lead to a maximum in contact pressure in the center of the polymer pin;
- The CoF mainly influences the contact pressure at the leading edge; the higher the CoF, the higher the maximal contact pressure at the leading edge. This effect could be minimized successfully in the disc setup presented in this paper.

The Poisson’s ratio and the CoF have a strong influence on the contact pressure distribution and their high values are assumed to have a critical influence on the reliability of the test conditions. Both values are typically high for polymers, which renders the tribological testing of polymers much more difficult. Based on the observations of the parameter study, a soft polyurethane was tested and characterized with a disc of 8 mm in diameter and 1 mm in height as sample geometry. From the generated data, a friction model was derived and implemented in an FEM model to replicate the experimental tests. Comparing the frictional output of one cycle of the experiments to the simulation showed good agreement, which verifies the accuracy of the model and the generation of reliable and robust data from the experimental testing procedure.

## Figures and Tables

**Table 1:** Model parameter for parameter variation.

Model Parameter	Symbol	Value ^1^				
Polymer sample height	h (mm)	0.25	0.5	**1.0**	1.5	2.0
Polymer sample diameter	d (mm)	4	6	**8**	12	16
Nominal contact pressure	$p_{n}$ (MPa)		0.5	**1.0**	2.0	4.0
Young’s modulus of polymer	E (MPa)		5	**10**	20	40
Poisson’s ratio of polymer	ν	0.0	0.3	**0.4**	0.49	0.499
CoF between polymer and counter body	μ	0.0	0.1	0.25	**0.5**	0.75

^1^ The values in bold indicate the base parameter set.

**Table 2:** Dimensionless parameters used in the parameter study.

Dimensionless Parameter	Formula
Normalized x-coordinate	$\;\hat{x}=\frac{2x}{d}$
Ratio of nominal contact pressure to Young’s modulus	${\;\hat{p}}_{n}=\frac{p_{n}}{E}$
Ratio of height to diameter	$\;\hat{p}=\frac{h}{d}$
Normalized contact pressure	${\;\hat{p}}_{c}=\frac{p_{c}}{p_{n}}$
Normalized contact area	${\;\hat{A}}_{c}=\frac{A_{c}}{A_{0}}$
Normalized mean contact pressure	${\;\hat{p}}_{c,m}=\frac{1}{A_{c}}\int_{A_{c}}{\;\hat{p}}_{c}\;\mathrm{dA}\;$

**Table 3:** Test matrix for tribological characterization of HPU premium.

Study No. ^1^	Loading Parameter	Value
1–2	Nominal contact pressure $p_{N}$ Average relative velocity $v_{\mathrm{rel}}$	1.0 MPa 3.0 MPa 50 mm/s 50 mm/s
3–4	Nominal contact pressure $p_{N}$ Average relative velocity $v_{\mathrm{rel}}$	1.0 MPa 3.0 MPa 100 mm/s 100 mm/s

^1^ Each study was performed with three repetitions.

**Table 4:** Model parameter for friction model.

Model Parameter	Symbol	Value
Static CoF	$\mu_{s}$	1.3
Dynamic CoF	$\mu_{d}$	0.9
Reference sliding velocity	$v_{0}$	25 mm/s
Reference contact pressure	$p_{0}$	1 MPa
Pressure exponent	n	0.26

**Table 5:** Input table for displacement over time for multilinear approximation of relative movement of counter surface.

Time (s)	Displacement (mm)
0.0	−27
0.03	−27
0.47	27
0.53	27
0.97	−27
1.0	−27

## Data Availability

Not applicable.
